# Intersectionality and women's empowerment in hysterectomy decisions: an inquiry using data from a large cross-sectional sample survey in India

**DOI:** 10.3389/fgwh.2025.1656684

**Published:** 2025-12-04

**Authors:** Anuj Kumar Pandey, Dyah Anantalia Widyastari, Benson Thomas M, Sajna Panolan, Pattraporn Chuenglertsiri, Bhubate Samutachak

**Affiliations:** 1 Institute for Population and Social Research, Mahidol University, Nakhon Pathom, Thailand; 2 Department of Health Management Research, International Institute of Health Management Research, New Delhi, India; 3 School of Public Health, SRM Institute of Science and Technology, Chennai, India

**Keywords:** hysterectomy, intersectionality, society, women empowerment, biological factors

## Abstract

**Background:**

Inspired by feminist theory and Durkheim's social perspective, this study used intersectionality to delve into the determinants of hysterectomy.

**Methods:**

Using data from the Demographic and Health Survey (DHS) of India, we examined the determinants of hysterectomy, focusing on three key themes: society, women's empowerment, and biological factors.

**Results:**

The overall hysterectomy rate in India increased from 31.5 per 1,000 women (age 15–49 years) during 2015–16 to 32.6 per 1,000 women during 2019–21. The results of bivariate and multivariate analyses echo the findings of the interaction analysis, indicating that, among women of the general caste, illiteracy and higher parity correlate with an increased likelihood of undergoing a hysterectomy. Illiterate women from the Other Backward Class also exhibited higher hysterectomy rates, regardless of parity. The second interaction result states that wealth influences hysterectomy, and illiteracy remains a significant risk factor across wealth statuses. The results of the third intersection indicate that higher education is a protective factor against hysterectomy, regardless of residence or parity.

**Conclusion:**

From the intersection of variables, the study observed that illiteracy, residing in rural areas, and high parity increase the likelihood of undergoing hysterectomy among women of reproductive age. There is a need to establish a mechanism for disseminating reproductive health knowledge to women in rural areas.

## Introduction

A hysterectomy is a common gynecological surgical procedure that involves the removal of the uterus. It is regularly performed worldwide, particularly after a cesarean section (1, 2). This life-saving procedure, on one side, saves the lives of women with deadly diseases such as carcinoma, fibroids, and severe postpartum hemorrhage. However, it can be harmful if performed in cases where it is not indicated (3). The World Health Organization (WHO) reported that approximately 1,540,000 women underwent hysterectomy globally in 2016 (4), with significant differences between high-income and low- and middle-income countries.

Available literature reports a declining trend in hysterectomies in developed countries owing to the availability of alternative medical procedures (5, 6). Conversely, there has been a significant increase in the hysterectomy cases in India, with geographical clustering (7–9). Studies have pointed to a skewed pattern of hysterectomies in India [11.35% of women ≥45 years, according to the Longitudinal Ageing Study in India (LASI) in 2018–19, and 3.2% of women of reproductive age, according to the Demographic and Health Survey (DHS) in 2015–16] (8, 10), concentrated in certain states/union territories. Evidence suggests significant spatial clustering in the prevalence of hysterectomy in India (10).

It is imperative that healthcare services be available to all, irrespective of religious beliefs, caste, region, religion, educational status, occupation, and much more. Women's health is influenced by several social factors (11, 12). Some studies have also reported that gender and healthcare-seeking among women are interrelated in many ways (2, 13). Studies have shown that there are gender-based differences with respect to decision-making regarding access to the appropriate treatment options (14). This gender-based empowerment requires further explanation in the Indian context (15).

Feminist theory has fostered inclusivity in society for women by recognizing their rights to reproductive and family planning choices, irrespective of social factors (16). While this theory examines the responsibilities and roles that women had in the past, it also explains these from social, political, and economic angles, thus providing ways to analyze gender inequality (17). Intersectionality theory recognizes and acknowledges the interconnectedness of race, gender, sexuality, class (18, 19), culture, the economy, power (18, 20), and other factors. These may have an influence when women make decisions regarding their health. Durkheim emphasized the significance of social variables such as religion, caste, and beliefs. He argued that humanity develops and survives within the framework of society; within this societal framework, religion serves as its primary refuge. Religion serves as a means of connecting people to build a community and providing social control, coherence, and shared purpose for individuals to engage with and reinforce social standards (21). These societal control mechanisms and established standards may contribute to the emergence of a gender-biased society. This contributes more to gender-related issues, such as discrimination and women's empowerment. It also affects women's decisions regarding their health, specifically the choice to undergo a hysterectomy, which can potentially limit their autonomy.

The primary indications for hysterectomy are leiomyomas, unexplained uterine hemorrhage, uterine prolapse, persistent pelvic pain, endometriosis, and malignant diseases. The decision to undergo a hysterectomy is influenced by the severity of the medical condition and is associated with several social elements, including women's empowerment and other societal concerns. Studies (7, 8, 22) have identified the determinants of hysterectomy. This study makes a unique contribution to addressing gaps in the existing literature. It assesses the prevalence and determinants of hysterectomy using an intersectional approach, considering the interplay between diverse biological and social factors. Understanding the intersectionality of social factors, women's empowerment, and decision-making with regard to hysterectomy involves recognizing how these factors interact and potentially reinforce or challenge the existing power dynamics (23, 24). It is necessary to explore how various identities and social categories intersect to shape a woman's experience and decision-making agency in the context of women's reproductive health.

## Methodology

**Figure 1:**
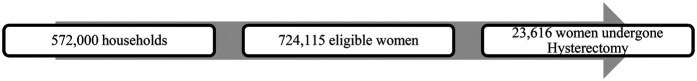
Survey sample included in the study, NFHS-5 | NFHS: national family health survey|.

We used publicly available data from the Demographic and Health Survey (DHS) of India. The DHS is also known as the National Family Health Survey (NFHS) and has been routinely conducted in India since 1991–92 (25). The NFHS is a nationally representative sample survey, and a dataset was available for the most recent survey, i.e., 2019–21. The NFHS employs a two-stage, stratified, random sampling method to make the results more representative of states and India as a whole. We used the data from the most recent round of the survey for in-depth analysis, while data from two rounds, i.e., 2015–16 and 2019–21, were used for a trend analysis of hysterectomy. The survey is based on robust sampling criteria, which makes the results more likely to be nationally representative. The most recent round of the survey achieved a response rate of 98%. As shown in Figure 1, a total of 724,115 women aged 15–49 were interviewed during the survey across the country, of whom 23,616 women underwent hysterectomy.

### Variables

The primary outcome of the study was hysterectomy, coded as “yes” or “no” for all 724,115 eligible women included in the survey. Explanatory variables were selected as proxies for the key themes identified as the determinants of hysterectomy, that is, society, women's empowerment, and biological factors. A detailed description of these variables is presented in Table 1. We conceptualized the assessment of an intersection of three sets of variables to provide a greater insight into women's biological parameters, a snapshot of the society in which they reside, and the crucial role of overall empowerment in decision-making and health-seeking behaviors. Caste, a principal variable for evaluating the societal theme as given by the theorist, had a 4.93% missing value in the DHS data; thus, a single imputation technique was used. Missing data were imputed based on baseline non-missing background characteristics, namely, place of residence (2, 26).

**Table 1:** Description and coding of the variables used to analyze the subcategories.

Variable name	Description and coding categories	Category under the study domain
Age of women	Coded as 0 “15–34” and 1 “35–49” based on risk factors	Biological factors
Parity (children ever born)	Coded as 0 for “Parity_1”, 1 for “Parity_2”, and 2 for “Parity_3+”.	Biological factors
Religion	Religion of the women was recoded as follows: Hindu, Muslim, Christian, Others	Society
Caste	Caste was recoded as follows: SC: Scheduled caste, ST: Scheduled tribe, OBC: Other backward class, and General	
Region	States were categorized into six regions: southern, central, northern, eastern, northeastern, and western. These were coded as 0, 1, 2, 3, 4, and 5, respectively.	
Place of residence	Urban and Rural	
Wealth Quintile	Recoded as: Poorest; poorer; middle; richer; richest; and high-wealth quintile.	
Age of respondent at first birth	Recoded as 0 for “Before 21 years of age” and 1 for “After 21 years of age"	Biological factors and women's empowerment
Decision-making power. (Cronbach's *α* score: 0.79)	Generated using a set of variables denoting “Person who usually decides how to spend the respondent’s earnings”, “Person who usually decides on large household purchases”, “Person who usually decides about the respondent's health care”, “Person who usually decides on visits to family or relatives”, and “Person who usually decides what to do with money husband earns”. Later recoded as: 0 for “ Less Autonomous” and 1 for “Autonomous”	Women's empowerment
Gender of head of household	Coded as Man and Woman. A total of 8 households with transgender heads of households were dropped before analysis.	
Having an A/C for oneself	Coded as Yes/No	
Owning a mobile phone	Coded as Yes/No	
Health insurance coverage	Coded as Yes/No	Women's empowerment
Media exposure (Cronbach α score: 0.47)	Generated using a set of variables denoting media exposure using variables such as “Reading newspapers or magazines”, “Listening to the radio”, “Watching television”. Later recoded as: 0 for “Not at all”, 1 for “Less than/at least once a week”	
Education	Women's educational attainment was recoded as: 0 = Illiterate; 1 = primary education; 2 = secondary education; 3 = higher and above	Women's empowerment

### Inclusion and exclusion criteria

The study included information from a total of 23,616 women who had undergone a hysterectomy. For the interaction analysis, as the sample size varied across selected covariates, only women with complete information on all selected covariates were included.

As shown in Table 1, a composite index was created using multiple nominal variables named the decision-making power (Cronbach *α* = .79), to explore the influence of women's decision-making ability on their likelihood of undergoing a hysterectomy. A total of 77,729 women provided information on the variables included in the decision-making process.

### Statistical analysis

Considering this objective, a trend analysis of frequency and distribution was undertaken over the past decade. The rate of hysterectomy was calculated for every 1,000 women of reproductive age, i.e., 15–49 years of age. The prevalence of hysterectomy per 1,000 women was also plotted geographically using QGIS (an open-source software) (27) to understand the state-wise distribution of hysterectomy in India. The causes of hysterectomy from two consecutive rounds of the NFHS were also examined. This analysis aimed to explore the association between the outcomes and explanatory variables. Bivariate analysis was conducted using the *χ*^2^ test for categorical variables. Variables identified as significant (*P* < .05) and those biologically plausible and aligned with the key themes identified as the determinants of hysterectomy, that is, society, women's empowerment, and biological factors, were selected for the adjusted analysis. The results of the regression analysis are presented as odds ratios (OR) with 95% confidence intervals.

Later, to assess the impact of the intersection of the key identified themes, an interaction analysis was undertaken using the proxy variables identified for each theme separately. Interaction refers to the unique characteristic of three or more variables, signifying that two or more variables collaboratively influence a third variable in a manner that is not additive (28). Only variables found to be significant with higher odds were included in the study of the intersection between biological factors and society and proxy variables for women's empowerment. The results are reported as odds ratios (ORs) with 95% confidence intervals.

### Ethics approval

All data used for the analysis are available in the public domain and were accessed after registration, declaring the purpose of the study. The used data is anonymized and publicly accessible. We adhered to the terms of use specified by the DHS Program Study and also adhered to the Declaration of Helsinki. Ethical approval for the NFHS surveys was obtained from the ethics review board of the International Institute for Population Sciences, Mumbai, India. These surveys were reviewed and approved by the ICF International Review Board. Informed written consent for participation in these surveys was obtained from the respondents during the surveys. Each individual's approval is sought before the patient interview, as per the consistent methodology followed in these national surveys.

### Results

**Figure 2:**
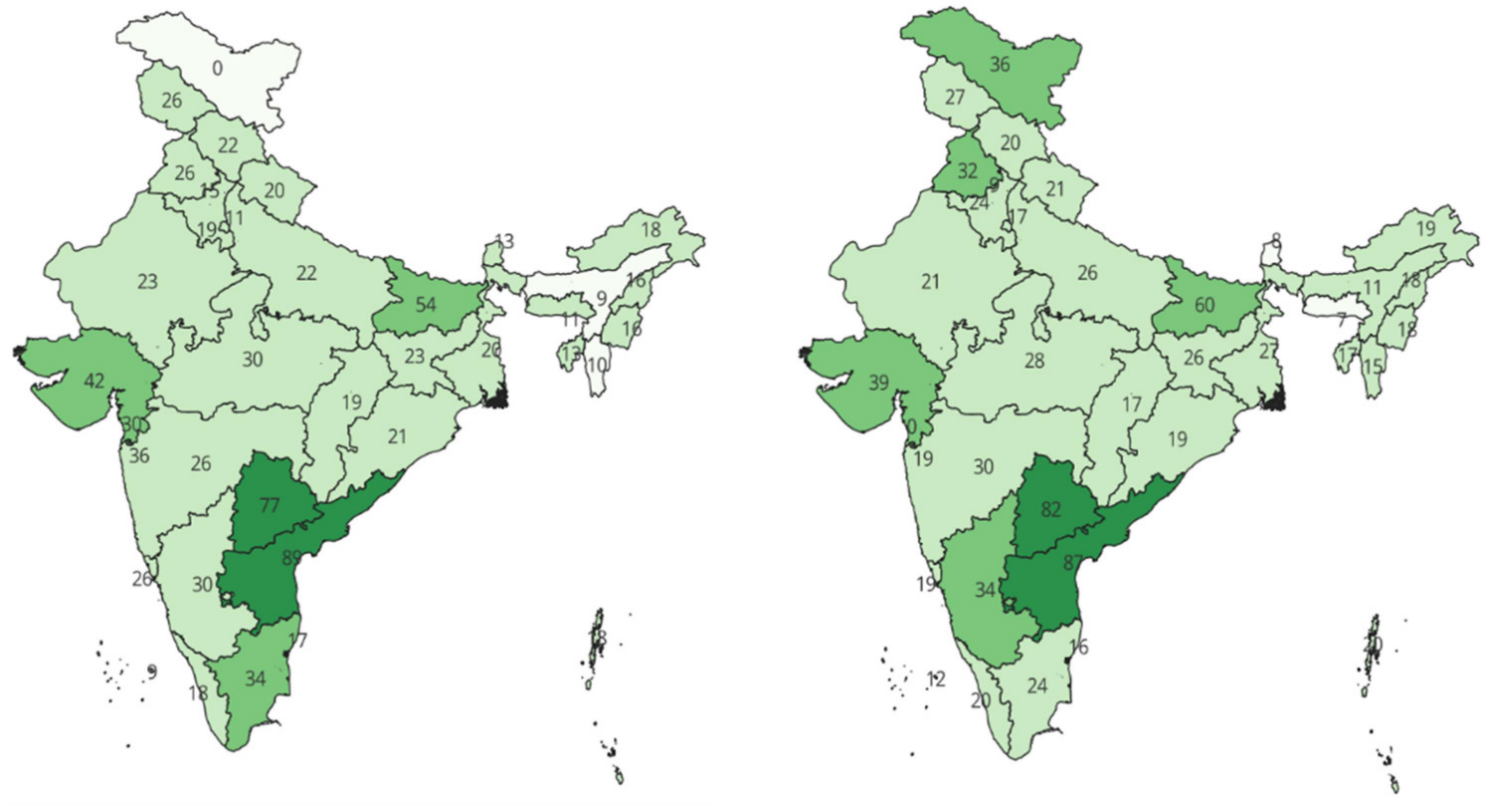
Hysterectomy procedures per 1,000 women aged 15–49 from 2015 to 16 (left) to 2019–21(right).

The overall hysterectomy rate in India slightly increased from 31.5 per 1,000 women (age 15–49 years) during 2015–16 to 32.6 per 1,000 women during 2019–21. Notably, there was a significant disparity in the prevalence of hysterectomy in India, ranging from 0 per 1,000 women in Daman and Diu to 87 per 1,000 women in Andhra Pradesh during 2019–21. There was also an increase in the regional disparity in access to hysterectomy procedures across India. Figure 2 provides the detailed prevalence of hysterectomy in each state/union territory (UT) in India. The figure also reveals substantial increases in the prevalence of hysterectomy in states such as Ladakh, Punjab, Bihar, Telangana, Karnataka, and Andhra Pradesh from 2015 to 16 to 2019–21.

**Figure 3:**
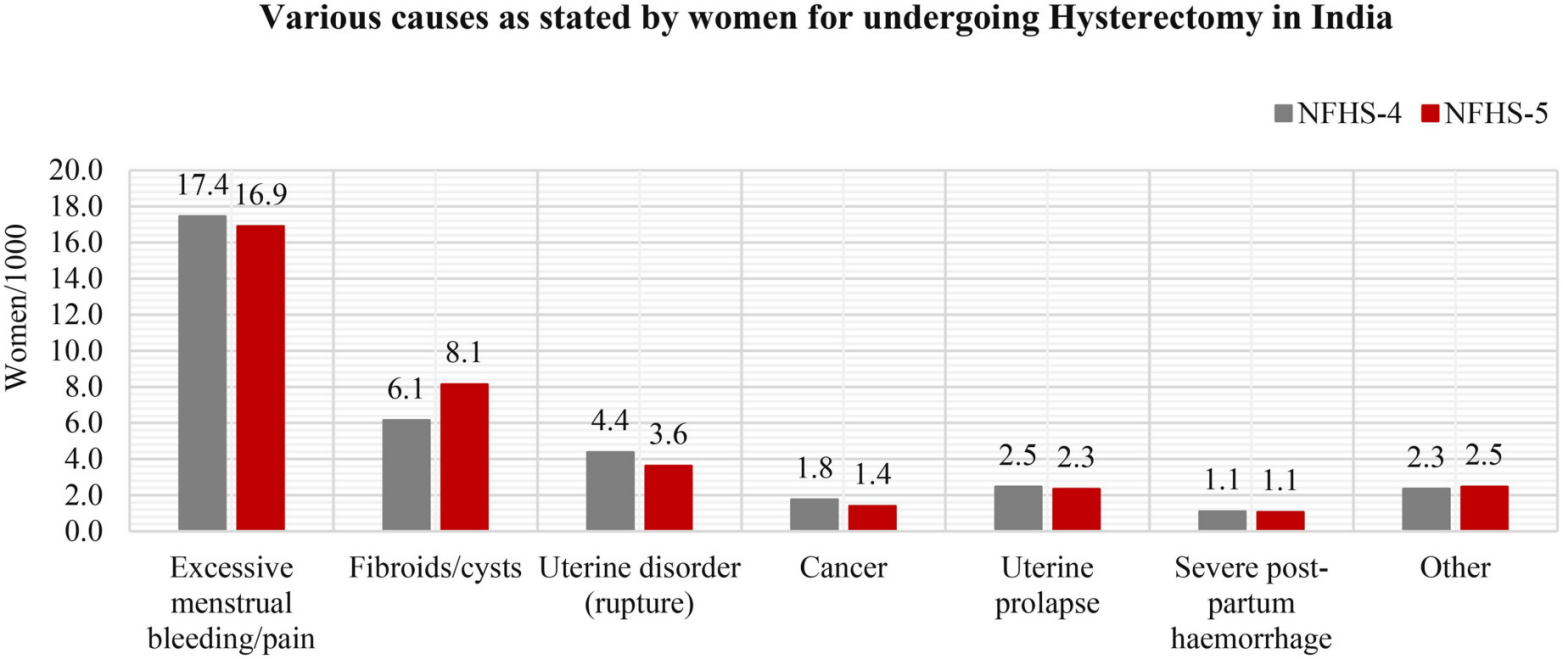
Causes of hysterectomy in two consecutive rounds of the NFHS. (**NFHS-4**: 2004-05; **NFHS-5**: 2015–16).

The majority of women reported that excessive menstrual bleeding was the cause of their hysterectomy. Fibroids were the second most common reason, with an increase of 8.1 per 1,000 women from 6.1 per 1,000 women from 2015 to 16 to 2019–21 (Figure 3).

Table 2 illustrates that the highest prevalence (78/1,000) was found among women aged 35 years. Respondents who belonged to the Hindu religion (34/1,000) and Other Backward Class (OBC) caste (36/1,000) had the highest prevalence of hysterectomy in India compared to other religions and castes. There was a significant difference in access to hysterectomy based on caste and religion, ranging from 22 to 23 per 1,000 women to 34–36 per 1,000 women. The prevalence of hysterectomy was also higher among those from rural areas (36/1,000) and those in the southern region of the country (46/1,000). The probability of having a hysterectomy was observed to increase if women have health insurance coverage (41/1,000).

From the univariate analysis, it is essential to note that illiteracy (72/1,000), no media exposure (42/1,000), lower age at first birth (58/1,000), and higher parity [parity +3 (66/1,000)] played crucial roles in deciding to undergo hysterectomy. Comparing proxy variables for women's empowerment, it was noted that having decision-making ability (50/1,000) and a bank account (34/1,000) also increased the probability of undergoing a hysterectomy (Table 2a).

**Table 2a:** Distribution of socio-demographic and health characteristics of women who underwent a hysterectomy.

Background Characteristics		# of Women who underwent a hysterectomy	# of Women (15–49 Y. O.)—No.	Hysterectomy per 1,000 women	(*P*-value)
Religion	Hindu	20,234	5,89,162	34	0.000
	Muslim	2,248	97,595	23	
	Christian	553	16,993	33	
	Others	581	20,359	29	
Caste	SC	5,311	1,73,198	31	0.000
	ST	1,635	73,715	22	
	OBC	11,517	3,20,913	36	
	General	5,153	1,56,284	33	
Region	Southern	6,763	1,48,076	46	0.000
	Central	4,700	1,86,292	25	
	Northern	1,289	52,000	25	
	Eastern	6,269	1,64,828	38	
	Northeastern	319	26,744	12	
	Western	4,276	1,46,168	29	
Place of Residence	Urban	5,988	2,35,275	26	0.000
	Rural	17,628	4,88,834	36	
Wealth Quintile	Poorest	3,820	1,33,973	29	0.000
	Poorer	5,049	1,44,812	35	
	Middle	5,483	1,48,613	37	
	Richer	5,206	1,50,680	35	
	Richest	4,057	1,46,031	28	
Health Insurance Coverage	No	14,742	5,08,597	29	0.000
	Yes	8,874	2,15,512	41	
Media Exposure	Not at all	6,847	1,62,700	42	0.000
	At least once a week	16,769	5,61,409	30	
Age of Women	15–34	2,876	4,59,507	6	0.000
	35–49	20,740	2,64,602	78	
Education	Illiterate	11,618	1,62,451	72	0.000
	Up to Secondary	11,155	4,48,314	25	
	Higher and above	843	1,13,345	7	
Parity (Children ever born)	Parity_1	2,168	3,26,286	7	0.000
	Parity_2	8,116	1,95,458	42	
	Parity_3+	13,332	2,02,365	66	
Age of respondent at first birth	20 and below	16,074	2,75,207	58	0.000
	21 and above	7,106	2,25,800	32	
Decision-making power	Less Autonomous	2,465	62,499	39	0.000
	Autonomous	740	14,927	50	
Gender of Head of Household^1^	Man	19,663	6,07,381	32	0.495
	Woman	3,952	1,16,729	34	
Having a bank account for oneself	No	641	23,154	28	0.000
	Yes	2,860	84,859	34	
Owning a Mobile Phone	No	1,844	49,744	37	0.000
	Yes	1,658	58,269	29	

Source: Authors' calculation from NFHS-5 survey data.

1 Transgender-6.

### Regression analysis

Logit regression analysis found that the likelihood of hysterectomy was significantly higher among women belonging to the OBC [1.18*** (1.14–1.22)], general caste [1.08*** (1.04–1.12)], and rural areas [1.43*** (1.39–1.48)]. Women belonging to the poor [1.23*** (1.18–1.28)] and middle [1.31*** (1.25–1.36)] wealth status also had higher odds of undergoing a hysterectomy (Table 2b).

**Table 2b:** Logistic regression analysis of determinants of hysterectomy.

Background Characteristics		Unadjusted OR (95% CI)	Adjusted OR (95% CI)
Religion	Hindu	Ref	Ref
	Muslim	0.66*** (0.63–0.69)	0.63*** (0.55–0.71)
	Christian	0.95 (0.87–1.03)	0.66*** (0.49–0.91)
	Others	0.83*** (0.76–0.90)	0.92 (0.71–1.20)
Caste	SC	Ref	Ref
	ST	0.72*** (0.68–0.76)	0.68*** (0.57–0.81)
	OBC	1.18*** (1.14–1.22)	1.42*** (1.28–1.56)
	General	1.08*** (1.04–1.12)	1.31*** (1.16–1.47)
Region	Southern	Ref	Ref
	Central	0.54*** (0.52–0.56)	0.73*** (0.65–0.82)
	Northern	0.53*** (0.50–0.56)	0.65*** (0.54–0.78)
	Eastern	0.83*** (0.80–0.86)	1.11* (0.99–1.25)
	Northeastern	0.25*** (0.22–0.28)	0.45*** (0.33–0.62)
	Western	0.63*** (0.61–0.65)	0.68*** (0.60–0.76)
Place of Residence	Urban	Ref	Ref
	Rural	1.43*** (1.39–1.48)	1.42*** (1.29–1.57)
Wealth Quintile	Poorest	Ref	Ref
	Poorer	1.23*** (1.18–1.28)	1.47*** (1.29–1.67)
	Middle	1.31*** (1.25–1.36)	1.73*** (1.51–1.98)
	Richer	1.22*** (1.17–1.27)	2.06*** (1.77–2.39)
	Richest	0.97 (0.93–1.02)	2.25*** (1.89–2.67)
Health Insurance Coverage	No	Ref	Ref
	Yes	1.44*** (1.40–1.48)	1.15*** (1.06–1.24)
Media Exposure	Not at all	Ref	Ref
	At least once a week	0.70*** (0.68–0.72)	0.97 (0.88–1.07)
Age of Women	15–34	Ref	Ref
	35–49	13.50*** (12.98–14.05)	6.40*** (5.70–7.18)
Education	Illiterate	Ref	Ref
	Up to Secondary	0.33*** (0.32–0.34)	0.60*** (0.55–0.65)
	Higher and above	0.10*** (0.09–0.10)	0.34*** (0.27–0.42)
Parity (Children ever born)	Parity_1	Ref	Ref
	Parity_2	6.48*** (6.18–6.80)	1.72*** (1.46–2.03)
	Parity_3+	10.55*** (10.07–11.04)	1.89*** (1.60–2.23)
Age of respondent at first birth	20 and below	Ref	Ref
	21 and above	0.52*** (0.51–0.54)	0.59*** (0.54–0.64)
Decision-making power	Less Autonomous	Ref	Ref
	Autonomous	1.27*** (1.17–1.38)	1.12** (1.03–1.23)
Gender of Head of Household^1^	Man	Ref	Ref
	Woman	1.05*** (1.01–1.08)	0.97 (0.86–1.09)
Having a bank account for oneself	No	Ref	Ref
	Yes	1.22*** (1.12–1.34)	0.96 (0.88–1.06)
Owning a Mobile Phone	No	Ref	Ref
	Yes	0.76*** (0.71–0.81)	0.93* (0.86–1.01)
			Constant 0.01*** (0.00–0.01)

Source: Authors' calculation from NFHS-5 survey data.

1 Transgender-6.

* *P*-value: <0.10.

** *P*-value: <0.05.

*** *P*-value: <0.001.

In contrast to all society-level variables, higher education [0.10*** (0.09–0.10)], media exposure [0.70*** (0.68–0.72)], and age at first birth if more than 21 [0.52*** (0.51–0.54)] also showed a protective effect. Having health insurance coverage increases the odds of hysterectomy [1.44*** (1.40–1.48)]. Comparing biological factors such as higher parity [10.55*** (10.07–11.04)] and age > 35 years [13.50*** (12.98–14.05)] increases the odds of having a hysterectomy manifold. Women with decision-making ability [1.27*** (1.17–1.38)], with women being the head of household [1.05*** (1.01–1.08)], and having a bank account [1.22*** (1.12–1.34)] also increased the odds of undergoing a hysterectomy. Table 2 also presents the statistics on the predictors of hysterectomy in India (Table 2b).

After adjusting for variables as per the identified themes and those found significant in the bivariate analysis, women residing in rural areas [1.42*** (1.29–1.57)], belonging to the richest wealth status [2.25*** (1.89–2.67)], being covered by health insurance [1.15*** (1.06–1.24)], having parity more than 3 [1.89*** (1.60–2.23)], and women with decision-making ability [1.12** (1.03–1.23)] had increased odds of hysterectomy. Higher literacy [0.34*** (0.27–0.42)] and exposure to media [0.97 (0.88–1.07)] decreased the odds of having a hysterectomy (Table 2b).

To comprehensively understand the outcomes arising from the intersection of the identified key themes, we employed interaction analysis to delve deeply into the connections and relationships between these identified themes. Interaction analysis driven by intersectionality theory also helps us understand that women as individuals have a combination of characteristics that may differ in their behavior (i.e., the decision to undergo a hysterectomy). The relative interaction effects of hysterectomy with the interaction between proxy variables indicating society, women's empowerment, and biological variables were estimated and are presented in Tables 3a–d. The intersection was assessed for key variables from each theme, and those were found with higher odds in the logit regression analysis. The intersection of key variables from each theme, namely Caste, Education, and Parity, as shown in Table 3a*,* which controlled for other essential variables, showed that illiterate women belonging to any caste and parity are more likely to undergo a hysterectomy than their counterparts. It is important to note that illiteracy had a greater influence on the odds of undergoing a hysterectomy, irrespective of caste or parity.

**Table 3a:** Results of the logit regression models for hysterectomy with the interaction between intersectionality covariates such as **caste, education, and parity**.

Caste	Education	Parity (Children ever born)		
		Parity_1	Parity_2	Parity_3+
SC	Illiterate	Ref.	1.85 (0.91, 3.8)	2.14** (1.08, 4.24)
	Up to Secondary	0.49 (0.21, 1.12)	0.97 (0.48, 1.97)	1.51 (0.76, 3.02)
	Higher and above	0.12** (0.02, 0.85)	0.52 (0.18, 1.45)	0.24 (0.03, 1.91)
ST	Illiterate	1.4 (0.49, 4)	1.42 (0.66, 3.05)	1.11 (0.54, 2.25)
	Up to Secondary	0.77 (0.29, 2.03)	0.79 (0.36, 1.74)	0.92 (0.43, 1.96)
	Higher and above	0.02 (0, 74.45)	0.02 (0, 14.05)	1.06 (0.13, 8.55)
OBC	Illiterate	2.81*** (1.34, 5.91)	3.46*** (1.74, 6.88)	2.54*** (1.29, 5)
	Up to Secondary	0.71 (0.34, 1.49)	1.5 (0.76, 2.97)	1.88 (0.95, 3.72)
	Higher and above	0.45 (0.18, 1.14)	0.81 (0.38, 1.74)	0.58 (0.2, 1.71)
General	Illiterate	2.2 (0.87, 5.56)	2.58*** (1.25, 5.34)	2.18** (1.09, 4.37)
	Up to Secondary	0.73 (0.33, 1.59)	1.44 (0.72, 2.87)	1.57 (0.79, 3.13)
	Higher and above	0.78 (0.33, 1.81)	1.01 (0.47, 2.16)	1.14 (0.44, 2.96)

Source: Authors' Calculation from NFHS-5 Survey Data.

Controlled for variables: Decision-making power, Gender of Head of Household, Age of respondent at first birth, having a bank account, having a mobile phone, age of women, religion, region, place of residence, wealth status, media exposure, and having health insurance.

* *P*-value: <0.10.

** *P*-value: <0.05.

*** *P*-value: <0.001.

Table 3a indicates that, within the general caste, women who are illiterate and have a higher number of children are more likely to undergo a hysterectomy. Illiterate women from the Other Backward Class (OBC), irrespective of the number of their children, are more likely to undergo a hysterectomy. Women in the scheduled caste who are better educated but have lower parity, or who are illiterate but have higher parity, are also more likely to undergo a hysterectomy. The intersection of caste-education-parity does not have any impact on the likelihood of women undergoing a hysterectomy.

A second attempt was made to assess the influence of wealth on the intersection of the identified variables within key themes, such as biological factors and women's empowerment. We noted that although a higher wealth status was found to significantly influence the likelihood of hysterectomy in the adjusted logit regression analysis, the intersection analysis revealed a different scenario. From Table 3b*,* it is evident that illiteracy plays a key role in determining the likelihood of hysterectomy. Although illiteracy remained non-significant in the majority of categories, it was a risk factor as indicated by the odds ratio. It also showed that illiterate women belonging to any wealth status and parity showed higher odds of hysterectomy. The data showed that, among the poorest women, lower parity and secondary education were protective factors against having a hysterectomy. Illiterate women and those with higher parity had a greater likelihood of undergoing a hysterectomy among women from the lower and medium socioeconomic classes. Irrespective of parity, illiteracy was found to be a risk factor for hysterectomy among wealthy women. Additionally, among wealthier women, lower parity and greater education are protective factors against hysterectomy. None of these indicators was significant among the wealthiest women.

**Table 3b:** Results of the logit regression models for hysterectomy with the interaction between intersectionality covariates such as wealth quintile, education, and parity.

Wealth status	Education	Parity (Children ever born)		
		Parity_1	Parity_2	Parity_3+
Poorest	Illiterate	Ref	1.29 (0.73, 2.3)	1.09 (0.65, 1.85)
	Up to Secondary	0.05*** (0.01, 0.42)	0.5** (0.26, 0.95)	1.02 (0.58, 1.78)
	Higher and above	#	0.81 (0.04, 15.1)	#
Poorer	Illiterate	0.91 (0.42, 1.96)	1.99** (1.14, 3.46)	1.6 (0.95, 2.71)
	Up to Secondary	0.34** (0.15, 0.76)	0.91 (0.52, 1.6)	1.12 (0.65, 1.93)
	Higher and above	#	0.16 (0.01, 2.43)	2.11 (0.68, 6.57)
Middle	Illiterate	1.73 (0.84, 3.57)	1.79** (1.03, 3.13)	2.01*** (1.19, 3.42)
	Up to Secondary	0.67 (0.35, 1.27)	1.13 (0.66, 1.95)	1.24 (0.72, 2.12)
	Higher and above	0.2 (0.03, 1.44)	0.77 (0.29, 2.09)	#
Richer	Illiterate	3.92*** (1.93, 7.94)	2.9*** (1.65, 5.08)	1.97** (1.15, 3.4)
	Up to Secondary	0.62 (0.33, 1.19)	1.29 (0.75, 2.21)	1.52 (0.89, 2.6)
	Higher and above	0.12** (0.03, 0.62)	0.59 (0.27, 1.29)	0.73 (0.25, 2.16)
Richest	Illiterate	#	2.71 (1.43, 5.13)	2.17 (1.23, 3.84)
	Up to Secondary	0.75 (0.39, 1.43)	1.23 (0.72, 2.13)	1.67 (0.97, 2.89)
	Higher and above	0.68 (0.35, 1.32)	0.85 (0.47, 1.53)	0.6 (0.25, 1.44)

Source: Authors' Calculation from NFHS-5 Survey Data.

#indicates no values in the intersection of the three key variables.

Controlled for variables: Decision-making power, Gender of Head of Household, Age of respondent at first birth, having a bank account, having a mobile phone, age of women, religion, region, place of residence, wealth status, media exposure, and having health insurance.

* *P*-value: <0.10.

** *P*-value: <0.05.

*** *P*-value: <0.001.

A third intersection was used to assess the influence of place of residence on the other two key theme variables (Table 3c). It is important to mention that education, even up to the secondary or higher level, has a higher protective influence on the likelihood of undergoing a hysterectomy, irrespective of place of residence and parity. Although illiteracy was found to be a risk factor, it was not statistically significant. The last intersection was analyzed to assess the influence of one of the key variables in women's empowerment themes in relation to education and parity. The results showed that, irrespective of autonomy in making decisions, education plays a key role in determining hysterectomy patterns (Table 3d).

**Table 3c:** Results of the logit regression models for hysterectomy with the interaction between intersectionality covariates such as residence, education, and parity.

Residence	Education	Parity (Children ever born)		
		Parity_1	Parity_2	Parity_3+
Urban	Illiterate	Ref.	1.07 (0.59, 1.92)	0.78 (0.45, 1.36)
	Up to Secondary	0.27*** (0.14, 0.5)	0.47** (0.27, 0.82)	0.68 (0.39, 1.17)
	Higher and above	0.23*** (0.12, 0.46)	0.27*** (0.15, 0.51)	0.23*** (0.09, 0.59)
Rural	Illiterate	1.11 (0.61, 2.02)	1.48 (0.86, 2.55)	1.29 (0.75, 2.21)
	Up to Secondary	0.35*** (0.19, 0.63)	0.74 (0.43, 1.27)	0.89 (0.52, 1.52)
	Higher and above	0.11*** (0.03, 0.34)	0.45** (0.24, 0.87)	0.42** (0.18, 0.97)

Source: Authors' Calculation from NFHS-5 Survey Data.

Controlled for variables: Decision-making power, Gender of Head of Household, Age of respondent at first birth, having a bank account, having a mobile phone, age of women, religion, region, place of residence, wealth status, media exposure, and having health insurance.

* *P*-value: <0.10.

** *P*-value: <0.05.

*** *P*-value: <0.001.

**Table 3d:** Results of the logit regression models for hysterectomy with the interaction between intersectionality covariates such as decision-making power, education, and parity.

Decision-making Power	Education	Parity (Children ever born)		
		Parity_1	Parity_2	Parity_3+
Less autonomous	Illiterate	Ref.	1.43** (1.03, 1.98)	1.22 (0.89, 1.66)
	Up to Secondary	0.35*** (0.24, 0.51)	0.72** (0.52, 0.98)	0.86 (0.63, 1.18)
	Higher and above	0.13*** (0.07, 0.27)	0.4*** (0.27, 0.61)	0.38*** (0.2, 0.73)
Autonomous	Illiterate	1.68 (0.99, 2.86)	1.61*** (1.11, 2.33)	1.35 (0.97, 1.89)
	Up to Secondary	0.39*** (0.23, 0.67)	0.71** (0.5, 1)	1.04 (0.74, 1.46)
	Higher and above	0.62 (0.34, 1.12)	0.45*** (0.26, 0.79)	0.35 (0.12, 1.04)

Source: Authors' calculation from NFHS-5 survey data.

Controlled for variables: Gender of head of household, age of respondent at first birth, having a bank account, having a mobile phone, age of women, religion, region, wealth status, media exposure, place of residence and having health insurance.

* *P*-value: <0.10.

** *P*-value: <0.05.

*** *P*-value: <0.001.

## Discussion

This study analyzed hysterectomy through three broad themes inspired by feminist theory and Durkheim's social perspective: society, women's empowerment, and biological factors. These variables were assessed in relation to each other to determine the factors that play a crucial role in influencing hysterectomy decisions in India. By incorporating this comparative lens, this study enriched its analysis, offering a broader perspective on the factors influencing hysterectomy prevalence across diverse sociocultural and healthcare contexts. This approach contributes to a more holistic understanding of the complex interplay among societal structures, women's autonomy, and biological determinants in shaping healthcare outcomes related to hysterectomy. Data from a nationally representative sample survey comprising 23,616 women who had undergone a hysterectomy, nested within 36 states/UTs and 707 districts, demonstrated a trivial increase in the prevalence of hysterectomy in India from 31.5 per 1,000 women (aged 15–49) during 2015–16 to 32.6 per 1,000 women during 2019–21. Similar to other studies, the maximum prevalence in hysterectomy was reported in southern (Telangana, Andhra Pradesh:< 50/1,000) and eastern India, followed by the central and western regions (10, 30, 31). The socioeconomic development of these regions, coupled with the presence of a large number of private healthcare facilities that promote unnecessary hysterectomies for financial gain, could explain the disparity in hysterectomy prevalence in the southern and eastern parts of India (10, 32). In line with this, other developed regions have also shown an increasing trend in age-specific hysterectomy rates (22, 33–36).

At the intersection of the key variables of all the identified themes, our study noted that illiteracy was a major factor in deciding to undergo a hysterectomy. The purpose of intersectionality theory is to challenge the notion of gender essentialism in feminism; that is, not all women experience the same plight. It is worth noting that all illiterate women, irrespective of social, biological, and empowerment level factors, are vulnerable to unwanted hysterectomy. The higher likelihood of hysterectomy among illiterate women could be due to a lack of reproductive health knowledge, such as no/limited media exposure. Knowledge about one's reproductive health protects women from engaging in harmful behavior. This includes poor menstrual hygiene practices and neglecting small signs such as white vaginal discharge, back pain, and more. These key findings are similar to those of another systematic review emphasizing the relevance of health literacy and adverse health outcomes (34, 37). The results of the intersection of key variables from all identified themes also indicated that illiteracy plays a crucial role in determining hysterectomy.

Further analysis of the intersection of caste, parity, wealth, and place of residence revealed that, although illiteracy played a crucial role in determining the surgical removal of the uterus, higher parity also increases the odds of hysterectomy, which has its own medical explanation in the fact that with each pregnancy, the likelihood of hysterectomy is higher. Although illiteracy and parity played pivotal roles in caste, illiterate women belonging to the OBC caste had the highest odds of undergoing a hysterectomy. Families belonging to the OBC caste usually fall into the middle and lower middle classes, where they are expected to have a reasonable amount of disposable income (38), and they usually run small businesses to support their families. This could explain the higher prevalence of hysterectomy among these women. In addition to socioeconomic status, healthcare development impacts the access of these communities to various preventive and alternative treatment options (39).

The results of the intersection analysis between wealth, education, and parity showed that the likelihood of hysterectomy is the highest among illiterate-rich women, regardless of parity, and lower among illiterate women from the poorest/poorer households. Hysterectomy odds were lower among women with higher education, regardless of SES status. This means that a woman's decision to undergo a hysterectomy depends on her knowledge of and ability to afford the procedure. In India, hysterectomy costs range from INR 4,124 to 57,622 (USD 54.98 to 768.29), including insurance coverage if eligible (40), implying that hysterectomy is most likely offered to wealthy families with little knowledge of the treatment. When women are more educated, although they can afford treatment, they will seek other treatment options and select hysterectomy only when their life is in danger. This result also aligns with the intersection of place of residence, education, and parity, where education served as a protective factor against undergoing a hysterectomy, irrespective of the area where the women lived (urban or rural).

The symbiotic relationship between literacy and autonomy is a testament to the interconnectedness of knowledge acquisition and individual agency, wherein education empowers individuals to exercise self-governance and autonomously make informed decisions. Our study echoes this same concept; self-autonomy was found to increase the odds of undergoing a hysterectomy, which is again higher among illiterate women who have decision-making power. We noted that while comparing the wealthiest illiterate women to the poorest (or urban/rural) illiterate women, the impacts of uterus removal are not the same, and autonomy plays a crucial role. The ability to assert autonomy over one's body is influenced by diverse factors, such as financial dependence and social circumstances, resulting in limited access to healthcare services or disregard for personal health.

Although having a bank account, another proxy variable considered under the empowerment theme, was found to be significantly associated with undergoing a hysterectomy, another similar variable, having a mobile phone, was not. Mobile availability in India can have a false effect on women's empowerment because of the relatively low cost of these phones. These findings corroborate those of other published studies (15, 41, 42). The intersection of these factors with other covariates, such as caste and religion, presents a picture portraying a society in which women do not need to be dependent on others for their health and knowledge of their own reproductive health. It is worth mentioning that an empowered woman is not only believed to bring down domination and oppression, but also brings down discrimination based on gender roles and opportunities. Age at first birth of less than 21 years and overall age > 35 years with higher parity also increased the odds of undergoing a hysterectomy. This suggests disparities in access to healthcare and socioeconomic inequality. Delaying medical care limits the chances of conservative treatment; thus, ultimately leading to hysterectomy in this section of society. Our analysis, irrespective of all other themes, shows that illiteracy played a crucial role in determining the likelihood of hysterectomy. Social media plays a crucial role in knowledge dissemination; our findings echo this concept. Mobile availability in India may not accurately reflect women's empowerment because of the cost of these phones. In this context, overall literacy stands out in our analysis, keeping back various biological, social, and other factors that influence women's empowerment.

Interestingly, hysterectomy is more common in rural areas (8, 10, 31). This draws attention to the fact that women in rural areas may have poorer reproductive health knowledge and are thus pressured by the private sector to undergo a hysterectomy. This was highlighted by a study conducted in Andhra Pradesh, with rural women deceived into undergoing a hysterectomy because of their illiteracy and vulnerability (43). The prevalence of hysterectomy was also found to be higher among women in rural areas and those belonging to the general castes. According to several studies (7, 22, 30), Muslims, a religious minority in India, have a lower prevalence of hysterectomies than Hindus and women of other religions. Delving deeper into this phenomenon, existing literature suggests that Muslim women have a reduced incidence of cervical cancer and human papillomavirus (HPV) infection, which are the primary contributors to the likelihood of undergoing a hysterectomy (44–46). Similarly, overall key variables in reproductive health showed that illiterate women were more likely to undergo a hysterectomy, implying a lack of awareness among uneducated women residing in rural areas in male-dominated families regarding reproductive choices and health-seeking behaviors. Knowledge of reproductive health plays a crucial role in determining overall well-being. These findings corroborate those of other published studies (47, 48).

The primary, self-reported reasons indicated for undergoing a hysterectomy in these studies are excessive menstrual bleeding, fibroids/cysts, uterine disorders, and uterine prolapse (8, 10, 31). However, while surgery proves to be lifesaving in some conditions, it can also be avoided by adopting alternative therapies (49). With the increasing rate of hysterectomies in India, the rational use of this procedure needs further exploration.

## Strengths and limitations

Using data from a substantial sample survey in India, encompassing a cohort of 724,115 eligible women, is a significant methodological strength of this work. Moreover, a strength of this study lies in its exploration of recent rounds of data and their subsequent analysis through the lens of two major sociological theories, thereby contributing to the existing body of literature on the determinants of hysterectomy. Another major strength is the comprehensive examination of biological, social, and women's empowerment factors as an intersection of each other. However, it is essential to acknowledge the limitations of this study. While sociological theories offer valuable insights into the social aspects of the condition, data from the DHS primarily focus on maternal and newborn health. Consequently, the available data, rooted in social origin, may provide limited information that reflects these theories. The available variables may not directly reflect the essence of Durkheim's feminist theory; nevertheless, we made an effort to analyze various proxy variables to provide a comprehensive picture of these theories. Additionally, determinants such as health insurance or decision-making autonomy may represent consequences rather than true causal factors, which could influence the interpretation of the findings.

## Conclusion

The study reiterates the increasing trend of hysterectomy in India, thus raising concerns. Illiteracy, residing in rural areas, and high parity increase the likelihood of undergoing a hysterectomy among women of reproductive age. There is a need to institute a mechanism for generating reproductive health knowledge among women to protect them from unwanted adverse outcomes, such as fibroids, excessive menstrual bleeding, and unwanted surgical removal of the uterus. Improving awareness of reproductive health needs among women in rural areas could prevent unnecessary hysterectomies in India and could avoid a potential financial burden on the country. The high rate of hysterectomies in underdeveloped communities is a concern. Medical illiteracy among women in rural communities needs urgent action to implement policies that inform and educate adolescent girls to improve their reproductive health knowledge. We further recommend the development of national guidelines on hysterectomy, applicable to both public and private facilities, to standardize clinical practices and ensure quality of care across regions.

Our findings underscore the role of social determinants in the overuse of hysterectomy, particularly among vulnerable populations. These insights can guide preventive strategies and inform future clinical research on conservative management and support the development of guidelines to reduce overtreatment.

## Data Availability

Publicly available datasets were analyzed in this study. This data can be found here: https://dhsprogram.com/data/.
